# Response to heterogeneity of tests and platforms in economic evaluations: synthetic model adoption; derivatives of transferable practice

**DOI:** 10.1017/S0266462323002787

**Published:** 2024-01-15

**Authors:** Benjamin Jones

**Affiliations:** School of Health Sciences, University of Southampton, Southampton, UK

With reference to:

Economic evaluation of genomic/genetic tests: A review and future directions: Published online by Cambridge University Press: 01 August 2022; Janet Bouttell, Robert Heggie, Karin Oien, Amy Romaniuk, Harper VanSteenhouse, Stephan von Delft, and Neil Hawkins.

Dear Colleagues,

As noted by Bouttell et al., 2022, at the time of publishing; attempts to resolve heterogeneity in economical evaluation continue to elude many parties. One proposition, drawn from carpentry, is put forth for community to discuss.

During the construction of oak-framed homes, the wood inevitably shapes and warps. To calculate the stresses and optimal positions for tenon and mortise joints, a hypothetical “perfect” line is mapped across the wood (plumb line). Calculation of angles and fit occur from this and the oak is then formed accordingly. This brings together imperfect items to work in their ideal manner, for example, natural bowing in material would have convexity face toward the sky to bear the load when used in a truss to absorb weight-bearing of the roof, whilst ignoring the evident imprecisions.

*Proposition*: Economic evaluation community should devise a model for optimal conditions irrespective of real-world constraints, for example, ignore local geography, legacy infrastructure, or select across the national picture for the most efficient components to create benchmark figures.

For pathology/sequencing laboratories that are resident/proximal to secondary care: Assume maximal efficiencies in delivery (or baseline of realism, for example, 1 hr logistical transport costs, semi-busy roads, median fuel costs). Expanding this illustration further; assume the lab design has no impediments to delivery, storage of materials/consumables are most cost-effective, configurations of assessment are premapped to standardised ideal (standardised operating procedure), all software platforms are assigned the lowest median cost to access/run, and all costs-per-sample are presumptively made upon “at-scale savings” and so forth. This model creates the theoretical ideal (plumb line) upon which all current real-world designs are benchmarked against, thus creating a figure that can assign a quantitative efficiency. One would assume all laboratories and healthcare providers to be inefficient compared to the ideal, but the extent (difference) could allow for an adjustment in calculation, that is, introduce handicap to modelling calculations.

Benefit of approach:

- Provides reflective questioning for current models existing in the real world, that is, comparison: Why are they so inefficient and what unique actions/processes/circumstance afford the most efficient approach?
- Allows for any model to factor the difference from a baseline calculation. Crudely put, the ideal model becomes the new zero and all measurements are now taken from this and not relative measurements against one another.

As such, we may find that the introduction of “genomic solutions” is indeed cost-effective, but not in the proposed host/current configuration. This would mitigate against the loss of those HTA inadvertently deemed “cost-ineffective” or assigned “uncertainty” by providing decision makers a contextual starting point:

The potential genomic solution may be both effective and economical **if** efficiencies at aforementioned key components (when contrasted against the proposed hypothetical model) are met within the newly submitted design or HTA assessment. No inefficiencies should exceed the median of most inefficient real-world providers.

